# Relugolix, an oral gonadotropin-releasing hormone (GnRH) receptor antagonist, in women with endometriosis-associated pain: phase 2 safety and efficacy 24-week results

**DOI:** 10.1186/s12905-021-01393-3

**Published:** 2021-06-21

**Authors:** Yutaka Osuga, Yoshifumi Seki, Masataka Tanimoto, Takeru Kusumoto, Kentarou Kudou, Naoki Terakawa

**Affiliations:** 1 Department of Obstetrics and Gynecology, Graduate School of Medicine, The University of Tokyo, 7 Chome-3-1 Hongo, Bunkyo City, Tokyo 113-0033 Japan; 2 Takeda Development Center Japan, Takeda Pharmaceutical Company Limited, Osaka, Japan; 3 Department of Obstetrics and Gynecology, Tottori University Faculty of Medicine, Yonago, Japan

**Keywords:** Relugolix, Endometriosis, Leuprorelin acetate, Extension study, Gonadotropin-releasing hormone antagonist

## Abstract

**Background:**

Relugolix is a once-daily, oral, nonpeptide, gonadotropin-releasing hormone receptor antagonist. The aim of this study was to evaluate safety of relugolix over 24 weeks in women with endometriosis-associated pain.

**Methods:**

This phase 2, randomized, open-label, parallel-group extension study was conducted in 101 clinics in Japan. Patients (premenopausal females ≥ 20 years) who completed the preceding 12-week relugolix phase 2 study continued to receive relugolix (10 mg, 20 mg, or 40 mg), placebo, or leuprorelin (3.75 mg) for an additional 12 weeks. Relugolix was administered orally once daily, and leuprorelin subcutaneously once every 4 weeks. The primary outcome was safety, including bone mineral density (BMD) and treatment-emergent adverse events (TEAEs). Secondary endpoints included visual analog scale (VAS) scores for endometriosis-associated pain. Analysis sets were defined as all patients who were administered the study drug.

**Results:**

Of 487 randomized patients in the preceding study, 397 enrolled in this extension study and continued to receive placebo (n = 77), relugolix 10 mg (n = 84), relugolix 20 mg (n = 78), relugolix 40 mg (n = 89), or leuprorelin (n = 69). Baseline characteristics were similar between extension study patients and patients in the preceding study. Frequency of TEAEs including metrorrhagia, menorrhagia, and hot flush was similar in the relugolix 40-mg and leuprorelin groups. Mean (SD) change in BMD from baseline at Week 24 was − 0.2 (1.99)% for placebo;  − 1.6 (2.34)%,  − 2.6 (2.94)%, and  − 4.9 (2.91)% for the relugolix 10-mg, 20-mg, and 40-mg groups, respectively; and − 4.4 (2.16)% for leuprorelin. Mean ± SD change from baseline in mean VAS score (mm) for pelvic pain at end of treatment was − 3.2 ± 12.16 for placebo; − 6.8 ± 10.56, − 9.0 ± 11.84, and − 11.9 ± 11.26 for the relugolix 10-mg, 20-mg, and 40-mg groups, respectively; and − 12.7 ± 12.57 for leuprorelin. Estradiol levels decreased with increasing relugolix dose and remained below postmenopausal levels throughout the 24-week relugolix 40-mg treatment period.

**Conclusions:**

Treatment with relugolix for 24 weeks was generally well tolerated and demonstrated similar pain reduction to leuprorelin in women with endometriosis. The dose-dependent loss in BMD observed with relugolix treatment was expected due to an induced hypoestrogenic state. Relugolix demonstrated a similar benefit/risk profile to injectable therapy in this phase 2 study.

*Trial registration* NCT01452685 (ClinicalTrials.gov, registered 17/10/2011).

**Supplementary Information:**

The online version contains supplementary material available at 10.1186/s12905-021-01393-3.

## Background

Women with endometriosis experience various clinical symptoms including pelvic pain, dysmenorrhea, dyspareunia, and infertility [1, 2]. Such symptoms substantially affect quality of life (QOL) in patients with endometriosis. The European Society of Human Reproduction and Embryology guideline recommends prescription of hormonal contraceptives, progestins, or gonadotropin-releasing hormone (GnRH) agonists as options for reducing endometriosis-associated pain [3]. However, oral contraceptive pills are associated with increased risk of thrombosis and hepatic dysfunction [4, 5], and there is limited evidence of their efficacy for endometriosis-associated pain [3, 6]. Progestins may induce abnormal bleeding [7, 8], and implant and depot injectable forms are associated with weight gain, nausea, and breast tenderness [8] and a decrease in bone mineral content [9]. Although GnRH agonists such as leuprorelin are highly effective in relieving endometriosis-associated symptoms, they decrease bone mineral content (due to an estrogen-lowering effect), which limits their use to less than 6 months without an add-back therapy [4]. In addition, GnRH agonists induce a transient increase in the secretion of gonadotropins (flare), which results in a temporary worsening of symptoms, and they cannot be orally administered [4].

An alternative therapeutic approach is the use of GnRH receptor antagonists, which do not induce an initial clinical flare and typically have fewer side effects than GnRH agonists [4]. Relugolix is an oral, nonpeptide GnRH receptor antagonist that reduces blood concentrations of hormones including estradiol (E_2_) and progesterone (P) via suppression of the hypothalamic–pituitary–gonadal (HPG) axis [10, 11], and induces endometrial atrophy. Therefore, relugolix is expected to improve the clinical symptoms of endometriosis, cause no flare, and have a faster onset of action than GnRH agonists. In Japanese premenopausal women with endometriosis, a phase 2 study (NCT01458301) evaluated ascending doses (10, 20, and 40 mg) of relugolix compared with placebo and demonstrated that doses up to 40 mg were well tolerated and effective in treating endometriosis-associated pain [12].

The present extension study (NCT01452685, registered 17/10/2011) evaluated the safety and efficacy of 12 additional weeks of therapy with relugolix (24 weeks in total) in patients who participated in the preceding phase 2 study.

## Methods

### Study design

This phase 2, multicenter, long-term extension study was conducted between March 2012 and February 2014 at 101 study sites in Japan. The study was designed as open-label, but study drug randomization information was only broken after testing and observation at Week 24 for the last patient in this study. The study was conducted in accordance with the principles of the Declaration of Helsinki, the International Council for Harmonisation Guideline for Good Clinical Practice, and all applicable laws and regulations. The protocol was reviewed and approved by the Institutional Review Boards at all participating study sites. All patients provided written informed consent before enrollment in this extension study.

The preceding study consisted of a pretreatment period of 4–12 weeks and a treatment period of 12 weeks; patients were randomized as previously described [12]. The present extension study consisted of an additional treatment period of 12 weeks and a follow-up period of 4 weeks (total period of study participation was 16 weeks). Overall treatment duration was 24 weeks, including the preceding study. Patients continued to receive the same treatment they were assigned at randomization in the preceding randomized controlled double-blind study (i.e., relugolix 10 mg, 20 mg, or 40 mg; placebo; or leuprorelin). To maintain blinding during the double-blind and extension phases, test results were concealed by the laboratory that conducted the drug concentration and pharmacodynamic analyses until the randomization schedule was broken.

Relugolix and its placebo were administered orally once daily 30 min before breakfast, and leuprorelin (3.75 mg) and its placebo were injected subcutaneously once every 4 weeks. Sex hormone preparations were prohibited during the study. A prescription analgesic (loxoprofen) was permitted when the investigator decided that an analgesic was required to alleviate severe endometriosis-associated pain. Analgesic use was recorded daily by the patients in their diaries.

Patients continuously recorded symptoms from the time of informed consent until after final study drug administration. Patients visited the investigational site every 4 weeks to undergo designated examinations and evaluations. During the follow-up period, if the first posttreatment menstruation was not observed by the final visit, the patient underwent further follow-up by telephone interview until the first posttreatment menstruation was observed.

### Patients

Eligible patients were premenopausal Japanese females who had completed treatment with the study drug in the preceding phase 2 study. Patients were excluded from the extension study if they met any of the following criteria: those who had treatment-emergent adverse events (TEAEs) in the preceding phase 2 study that made study drug continuation not in the best interest of the patient; those unable to comply with the study protocol requirements due to the development of a new disease or symptom, or aggravation of clinical laboratory findings; those for whom treatment showed no efficacy during the preceding phase 2 study; and those who had problems with continuing the study because of the occurrence of symptoms of hypoestrogenism.

**Fig. 1:**
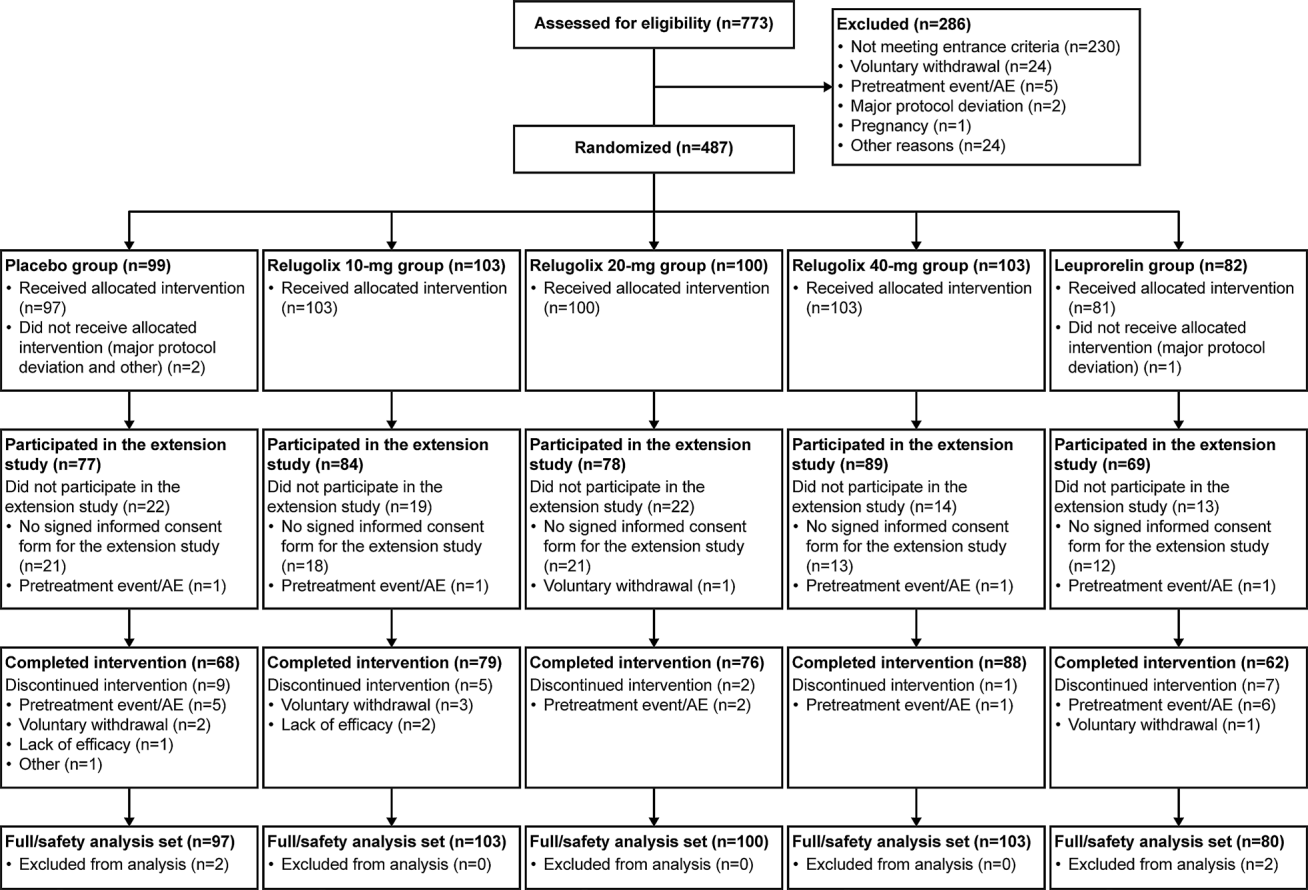
Patient flowchart. *AE* adverse event

In the preceding phase 2 study, 487 patients were randomized to treatment groups and 484 patients were administered the study drug. Of these patients, 402 (83.1%) signed the informed consent form to participate in this extension study. Of the 402 patients, five did not participate in the extension study for the following reasons: pretreatment event/adverse event in four patients and voluntary withdrawal in one patient (Fig. 1). Therefore, 397 patients were enrolled in the extension study and administered the study drug: 77 patients in the placebo group, 84 in the relugolix 10-mg group, 78 in the relugolix 20-mg group, 89 in the relugolix 40-mg group, and 69 in the leuprorelin group. The FAS/SAS for the 24 weeks of study drug administration included 483 patients (Fig. 1) because three patients were not administered the study drug and one patient had a major violation of Good Clinical Practice (loss of source documents).

### Study variables

The primary endpoints in this study were assessments of safety, including bone mineral density (BMD) assessed by dual energy X-ray absorptiometry, TEAEs, vital signs, weight, 12-lead electrocardiogram (ECG), and clinical laboratory tests. Secondary endpoints included visual analog scale (VAS) scores for pelvic pain, dysmenorrhea, and dyspareunia during the treatment period. Additional endpoints included endometriosis-associated pain symptoms assessed by modified Biberoglu and Behrman (M-B&B) and B&B scales during the treatment period [13]; use of analgesics during the treatment period; decrease in menstrual blood loss (based on self-reported amount of bleeding scores); achievement of amenorrheic state; QOL assessed by Endometriosis Health Profile-30 (EHP-30) [14, 15]; and blood concentration of E_2_, P, luteinizing hormone (LH), and follicle-stimulating hormone (FSH).

### Statistical analyses

Data from the preceding phase 2 study were combined with data from the present extension study to analyze safety, efficacy, and pharmacodynamics across 24 weeks of relugolix administration. For this reason and consistent with the preceding study, the number of evaluable patients was set at 450.

In the present extension study, the full analysis set (FAS) and safety analysis set (SAS) were the same, defined as all patients who received at least one dose of the study drug in the preceding phase 2 study. TEAEs were coded using the Medical Dictionary for Regulatory Activities Version 16.1. For continuous variables (including BMD, vital signs, weight, clinical laboratory tests, and ECG), baseline values, observed values, and changes from baseline were summarized for each measurement time point. For the secondary efficacy endpoints, summary statistics and 95% confidence intervals were calculated for each treatment group.

## Results

The mean VAS score (mm) for pelvic pain, dysmenorrhea, and dyspareunia at baseline for patients who entered the extension study ranged from 14.6 to 16.0, 26.6 to 31.57, and 8.1 to 12.6, respectively. Overall, there were no apparent differences in demographic and baseline characteristics among the treatment groups (Additional file 1). There were no clinically important differences in demographic and baseline characteristics between patients randomized in the preceding phase 2 study [12] and the subgroup who entered the extension study.

### Safety assessment

The incidences of TEAEs over 24 weeks were 81.4% in the placebo group, 86.4% in the relugolix 10-mg group, 96.0% in the relugolix 20-mg group, 95.1% in the relugolix 40-mg group, and 97.5% in the leuprorelin group (Table 1). The incidences of TEAEs in the relugolix 20-mg and 40-mg groups were higher compared with the placebo group, and similar to that in the leuprorelin group. TEAEs with an incidence of ≥ 10% in any relugolix group were nasopharyngitis, headache, metrorrhagia, menstruation irregular, menorrhagia, oligomenorrhea, hyperhidrosis, and hot flush (Table 1). All TEAEs were mild or moderate in intensity except for two severe events (blood creatine phosphokinase increased and ovarian cyst ruptured) in the placebo group.

**Table 1:** Summary of TEAEs

	Relugolix			Leuprorelin (n = 80)	Placebo (n = 97)
	10 mg (n = 103)	20 mg (n = 100)	40 mg (n = 103)		
Number of TEAEs	334	365	407	334	263
Patients with any TEAEs	89 (86.4)	96 (96.0)	98 (95.1)	78 (97.5)	79 (81.4)
Patients with drug-related TEAEs	68 (66.0)	88 (88.0)	91 (88.3)	72 (90.0)	38 (39.2)
*Intensity of TEAEs*					
Mild	83 (80.6)	82 (82.0)	83 (80.6)	64 (80.0)	68 (70.1)
Moderate	6 (5.8)	14 (14.0)	15 (14.6)	14 (17.5)	9 (9.3)
Severe	0 (0.0)	0 (0.0)	0 (0.0)	0 (0.0)	2 (2.1)
TEAEs leading to study drug discontinuation	1 (1.0)	7 (7.0)	2 (1.9)	9 (11.3)	6 (6.2)
Serious TEAEs	0 (0.0)	2 (2.0)	0 (0.0)	0 (0.0)	5 (5.2)
*TEAEs occurring in* ≥ *10% of patients in any treatment group*					
Nasopharyngitis	31 (30.1)	31 (31.0)	31 (30.1)	26 (32.5)	32 (33.0)
Headache	5 (4.9)	12 (12.0)	11 (10.7)	11 (13.8)	10 (10.3)
Metrorrhagia	28 (27.2)	36 (36.0)	30 (29.1)	32 (40.0)	8 (8.2)
Menstruation irregular	21 (20.4)	21 (21.0)	7 (6.8)	5 (6.3)	5 (5.2)
Menorrhagia	11 (10.7)	16 (16.0)	15 (14.6)	9 (11.3)	5 (5.2)
Oligomenorrhea	12 (11.7)	12 (12.0)	1 (1.0)	0 (0.0)	2 (2.1)
Genital hemorrhage	3 (2.9)	5 (5.0)	7 (6.8)	8 (10.0)	2 (2.1)
Hyperhidrosis	4 (3.9)	11 (11.0)	10 (9.7)	10 (12.5)	1 (1.0)
Hot flush	12 (11.7)	23 (23.0)	55 (53.4)	37 (46.3)	8 (8.2)

Data are n (%), unless otherwise stated

*TEAE* treatment-emergent adverse event

There were no deaths, and seven serious TEAEs were reported throughout the preceding and extension studies, including Langerhans’ cell histiocytosis, narcolepsy, ovarian cyst ruptured (two patients), and hemorrhagic ovarian cyst in the placebo group, and pseudocyst and liver function test abnormal in the relugolix 20-mg group. Of these, three serious TEAEs (hemorrhagic ovarian cyst and ovarian cyst ruptured in the placebo group, and pseudocyst in the relugolix 20-mg group) were observed during the extension study. An abnormal liver function test was reported in one patient in the relugolix 20-mg group in the preceding study; this was considered related to the study drug and resolved after study drug discontinuation. The incidences of TEAEs leading to discontinuation of the study drug were 6.2% in the placebo group, 1.0% in the relugolix 10-mg group, 7.0% in the relugolix 20-mg group, 1.9% in the relugolix 40-mg group, and 11.3% in the leuprorelin group. No clinically significant changes were found in clinical laboratory test results, vital signs, or ECG findings in the extension study.

The mean percent changes in BMD from baseline at the lumbar spine (L1–L5) at Week 12 and 24 (SD) were − 0.1 (1.73)% and − 0.2 (1.99)%, respectively, for placebo, − 1.0 (1.88)% and − 1.6 (2.34)% for relugolix 10 mg, − 1.3 (2.09)% and − 2.6 (2.94)% for relugolix 20 mg, − 2.1 (2.22)% and − 4.9 (2.91)% for relugolix 40 mg, and − 2.2 (1.67)% and − 4.4 (2.16)% for leuprorelin. The decrease in BMD was time- and dose-dependent in the relugolix groups. The percent change from baseline in BMD in the relugolix 40-mg group was similar to that in the leuprorelin group. As for TEAEs related to BMD, 14 patients with BMD decreases at Week 24 [placebo (n = 1), relugolix 10 mg (n = 1), relugolix 20 mg (n = 2), relugolix 40 mg (n = 6), and leuprorelin (n = 4)] and two patients at Week 12 [relugolix 20 mg (n = 1) and 40 mg (n = 1)] were reported. All these TEAEs were considered related to the study drug and, except for one moderate event in the leuprorelin group, all the TEAEs related to BMD were considered mild in intensity by the investigator.

In the follow-up period, the return of menstrual cycles was not confirmed in 24 out of 483 patients for the following reasons: start of treatment with hormone preparations prior to the return of menstrual cycle (n = 19); surgical operation (n = 2); pregnancy (n = 1); lost to follow-up (n = 1); and the decision by the investigator that follow-up was not necessary (n = 1). The mean duration from the last dose of study drug to the return of menstrual cycles (SD) was 17.3 (8.49) days for placebo, 21.0 (12.32) days for relugolix 10 mg, 26.0 (12.97) days for relugolix 20 mg, 36.9 (9.49) days for relugolix 40 mg, and 73.3 (21.11) days for leuprorelin.

### Efficacy assessments

**Fig. 2:**
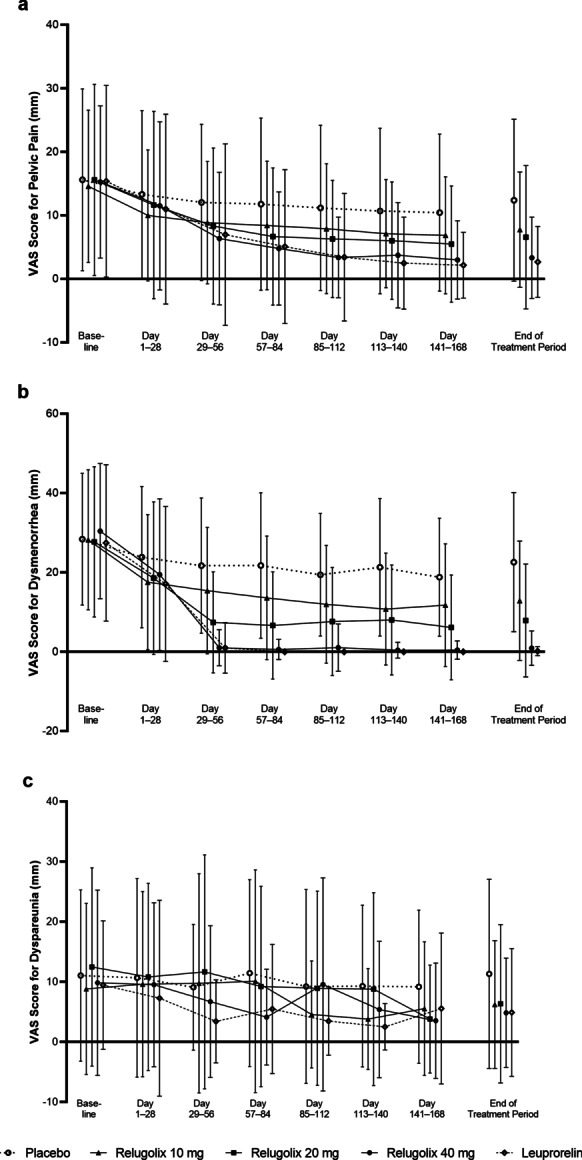
VAS score (mm) by visit. **a** Pelvic pain (end-of-treatment group sizes: placebo n = 97, relugolix 10 mg n = 103, relugolix 20 mg n = 100, relugolix 40 mg n = 103, leuprorelin n = 80). **b** Dysmenorrhea (end-of-treatment group sizes: placebo n = 97, relugolix 10 mg n = 103, relugolix 20 mg n = 100, relugolix 40 mg n = 103, leuprorelin n = 80). **c** Dyspareunia (end-of-treatment group sizes: placebo n = 36, relugolix 10 mg n = 50, relugolix 20 mg n = 40, relugolix 40 mg n = 39, leuprorelin n = 23). Data are mean ± SD. *SD* standard deviation; *VAS* visual analog scale

The VAS scores for pelvic pain and dysmenorrhea in the relugolix and leuprorelin groups decreased in a time- and dose-dependent manner throughout the treatment period (Fig. 2a, b). No clear trend was observed in mean VAS score from baseline for dyspareunia among the treatment groups (Fig. 2c). The mean changes from baseline to end of the treatment period in mean VAS score (mm) for pelvic pain, dysmenorrhea, and dyspareunia (SD) were − 3.2 (12.16), − 5.8 (17.13), and − 1.1 (12.66), respectively, for placebo; − 6.8 (10.56), − 15.4 (18.05), and − 3.5 (10.85), respectively, for relugolix 10 mg; − 9.0 (11.84), − 19.8 (20.43), and − 3.6 (11.55), respectively, for relugolix 20 mg; − 11.9 (11.26), − 29.5 (17.54), and − 0.9 (12.04), respectively, for relugolix 40 mg; and − 12.7 (12.57), − 27.2 (19.86), and − 4.6 (15.09), respectively, for leuprorelin. The changes in mean VAS score from baseline for pelvic pain and dysmenorrhea in the relugolix 40-mg group were similar to those in the leuprorelin group. Overall, similar results were obtained in mean M-B&B (Additional file 2) and B&B (Additional file 3) scores for pelvic pain, dysmenorrhea, and dyspareunia.

The change from baseline in proportion of days with analgesic use showed a time- and dose-dependent decrease in the relugolix groups compared with placebo (Additional file 4). Dose-dependent improvements in QOL at Weeks 12 and 24 were demonstrated by changes in the EHP-30 scores in the “pain” (reflecting on daily activities and functioning) and “control and powerlessness” domains (Additional file 4). Patients receiving relugolix and leuprorelin had greater improvements in these QOL domains than patients receiving placebo.

Relugolix, at higher doses (20 mg and 40 mg), was associated with lower median values of E_2_, LH, FSH, and P (Additional file 5). In the relugolix 40-mg group, the median serum E_2_ concentration decreased to < 10 pg/mL (less than the lower limit of quantification [LLQ]) at Week 2 and was maintained at this level until Week 24; in contrast, in the leuprorelin group, the median serum E_2_ concentration did not decrease to LLQ until Week 4.

## Discussion

In this phase 2 extension study in Japanese women with endometriosis, pelvic pain and dysmenorrhea decreased in a time- and dose-dependent manner over 24 consecutive weeks of treatment with relugolix (up to 40 mg). The majority of the TEAEs experienced by patients treated with relugolix were mild, and TEAEs leading to study drug discontinuation were uncommon. These safety and efficacy results over 24 weeks were consistent with the findings from the first 12 weeks of the phase 2 study [12].

There were no unexpected safety issues during the extended administration period of 12 weeks. Of the TEAEs reported, most were reported during the first 12 weeks [12]. The overall incidence of TEAEs was higher in the relugolix 20-mg and 40-mg groups compared with the placebo group, but was similar to the leuprorelin group. The most common TEAEs in the relugolix group included nasopharyngitis, metrorrhagia, irregular menstruation, menorrhagia, oligomenorrhea, hyperhidrosis, headache, and hot flush. These TEAEs were observed at similar frequencies in the leuprorelin group and were consistent with the estrogen-lowering effects of both relugolix and leuprorelin. In addition, relugolix time- and dose-dependently decreased BMD, with the change in BMD in the relugolix 40-mg group being similar to that in the leuprorelin group. Again, this TEAE was considered to be secondary to the hypoestrogenic state induced by relugolix and leuprorelin. These hypoestrogenic side effects may potentially be mitigated through add-back therapy using low-dose hormones, an approach that has been used with GnRH agonists such as leuprorelin [16] and is under investigation in ongoing global phase 3 studies of relugolix (ClinicalTrials.gov: NCT03049735, NCT03103087, NCT03204318, NCT03204331, NCT03654274).

Following cessation of the study drug, the return of menstrual cycles was confirmed in the majority of patients. The number of days until recovery of menstruation in the relugolix 40-mg group was approximately half of that in the leuprorelin group. This was considered to be related to a difference in the timing of HPG axis recovery after removal of an oral GnRH antagonist (relugolix) versus an injectable depot formulation of an agonist (leuprorelin). This is supported by the pharmacodynamic data: after suppression of E_2_ in the relugolix 40-mg and leuprorelin groups throughout the study period, E_2_ levels recovered during the follow-up period in the relugolix 40-mg group but remained low in the leuprorelin group.

Overall, in patients with endometriosis, the effects of relugolix on endometriosis-related pain after administration for 12 weeks in the preceding phase 2 study [12] were maintained for an additional 12 weeks. VAS scores for pelvic pain and dysmenorrhea in the relugolix and leuprorelin groups time- and dose-dependently decreased throughout the treatment period and were lower than placebo. VAS scores in the relugolix 40-mg group were similar to those in the leuprorelin group. These efficacy results were supported by the results for other pain evaluation indexes, M-B&B and B&B score, and the time- and dose-dependent reduction in the proportion of patients using analgesics in the relugolix groups. Although direct comparisons of efficacy between studies can be difficult owing to differences in pain rating scales, the efficacy of relugolix for reducing endometriosis-related pain is consistent with the findings for elagolix, another oral GnRH antagonist [17]. Other endometriosis-related symptoms were also time- and dose-dependently improved by treatment with relugolix. The amount of menstrual bleeding decreased, the proportion of patients who achieved amenorrhea increased, and QOL was improved in the “pain” and “control and powerlessness” domains of the EHP-30. These results suggest that relugolix improves more clinical symptoms than just endometriosis-related pain.

The main strength of this study is the extended period of treatment, which enabled further elucidation of the safety and efficacy of relugolix beyond the initial 12-week phase 2 study period. Limitations of this study included a relatively small number of patients entering the extension period and the inclusion of only Japanese patients, both of which may reduce the generalizability of the results. Furthermore, the number of patients who received the assessment for dyspareunia was small in the present study, so the efficacy of relugolix for dyspareunia remains to be confirmed in larger studies.

## Conclusions

In conclusion, treatment with relugolix up to 40 mg for 24 weeks improved endometriosis-related pain and was generally well tolerated. Relugolix 40 mg demonstrated similar efficacy to leuprorelin in premenopausal women with endometriosis. Relugolix may be a new oral treatment option for endometriosis-associated pain that has a similar benefit/risk profile to injectable therapy, without the initial hormonal flare and a more rapid return of menses after treatment discontinuation. The 40-mg dose of relugolix is currently under evaluation in phase 3 studies of patients with endometriosis.

## Supplementary Information

**Additional file 1.** Demographic and baseline characteristics.

**Additional file 2.** Change from baseline in mean of M-B&B score for pelvic pain, dysmenorrhea, and dyspareunia by visit.

**Additional file 3.** Change from baseline in mean of B&B score for pelvic pain, dysmenorrhea, and dyspareunia by visit.

**Additional file 4.** Results of other endpoints.

**Additional file 5.** Summary of pharmacodynamic parameters at each assessment point.

## Data Availability

The datasets, including the redacted study protocol, redacted statistical analysis plan, and individual participants data supporting the results reported in this article, will be made available within three months from initial request, to researchers who provide a methodologically sound proposal. The data will be provided after its de-identification, in compliance with applicable privacy laws, data protection and requirements for consent and anonymization. For details on submitting a request, see the instructions provided at https://clinicaltrials.takeda.com/.

## Abbreviations

B&B: Biberoglu and Behrman (scale)
BMD: Bone mineral density
E_2_: Estradiol
ECG: Electrocardiogram
EHP-30: Endometriosis Health Profile-30
FAS: Full analysis set
FSH: Follicle-stimulating hormone
GnRH: Gonadotropin-releasing hormone
HPG: Hypothalamic–pituitary–gonadal
LH: Luteinizing hormone
LLQ: Lower limit of quantification
M-B&B: Modified Biberoglu and Behrman (scale)
P: Progesterone
QOL: Quality of life
SAS: Safety analysis set
TEAE: Treatment-emergent adverse event
VAS: Visual analog scale
