# Supplementary material for: Relugolix, an oral gonadotropin-releasing hormone (GnRH) receptor antagonist, in women with endometriosis-associated pain: phase 2 safety and efficacy 24-week results
Source: BMC Womens Health. 2021 Jun 21;21:250. doi: 10.1186/s12905-021-01393-3 (PMC8218467; doi:10.1186/s12905-021-01393-3)
Supplement: Supplementary file 1 — Additional file 1. Demographic and baseline characteristics. [file 12905_2021_1393_MOESM1_ESM.docx]

**Additional Files**

**Additional file 1** Demographic and baseline characteristics

| Characteristic | Relugolix | | | Leuprorelin  (n=69) | Placebo  (n=77) | Total  (N=397) |
| --- | --- | --- | --- | --- | --- | --- |
|  | 10 mg  (n=84) | 20 mg  (n=78) | 40 mg  (n=89) |  |  |  |
| Age, years, mean (SD) | 35.3 (6.41) | 35.3 (7.01) | 35.4 (6.15) | 36.6 (6.14) | 35.9 (5.99) | 35.7 (6.34) |
| BMI, kg/m^2^, mean (SD) | 21.2 (3.08) | 20.5 (2.54) | 21.5 (3.18) | 21.7 (3.34) | 21.2 (2.88) | 21.2 (3.03) |
| Disease duration, years, mean (SD) | 3.6 (5.04) | 3.4 (3.95) | 4.1 (5.35) | 3.1 (3.97) | 3.8 (4.69) | 3.6 (4.67) |
| VAS score (mm) at baseline, mean (SD) | | |  |  |  |  |
| Pelvic pain | 14.6 (12.72) | 14.8 (13.99) | 15.8 (12.50) | 16.0 (15.88) | 15.0 (13.92) | 15.3 (13.69) |
| Dysmenorrhea | 26.9 (17.15) | 26.6 (18.64) | 31.6 (17.44) | 28.4 (20.35) | 28.1 (15.95) | 28.4 (17.89) |
| Dyspareunia | 8.1 (15.20)  (n=36) | 13.5 (17.06)  (n=38) | 9.2 (14.25)  (n=33) | 10.4 (11.25)  (n=22) | 12.6 (15.21)  (n=31) | 10.8 (14.98)  (n=160) |
| M-B&B score at baseline, mean (SD) | |  |  |  |  |  |
| Pelvic pain | 0.7 (0.48) | 0.6 (0.45) | 0.7 (0.45) | 0.7 (0.56) | 0.6 (0.46) | 0.7 (0.48) |
| Dysmenorrhea | 1.1 (0.45) | 1.2 (0.49) | 1.2 (0.47) | 1.2 (0.48) | 1.1 (0.45) | 1.2 (0.47) |
| Deep dyspareunia | 0.5 (0.60)  (n=36) | 0.7 (0.57)  (n=38) | 0.6 (0.47)  (n=33) | 0.6 (0.44)  (n=22) | 0.6 (0.43)  (n=31) | 0.6 (0.51)  (n=160) |
| B&B score at baseline, mean (SD) | |  |  |  |  |  |
| Pelvic pain | 1.7 (0.68) | 1.6 (0.63) | 1.5 (0.59) | 1.6 (0.57) | 1.6 (0.55) | 1.6 (0.61) |
| Dysmenorrhea | 2.1 (0.46) | 2.1 (0.47) | 2.1 (0.47) | 2.1 (0.46) | 2.0 (0.38) | 2.1 (0.45) |
| Dyspareunia | 0.7 (0.67)  (n=37) | 1.0 (0.73)  (n=40) | 0.6 (0.55)  (n=33) | 0.9 (0.75)  (n=22) | 0.9 (0.68)  (n=31) | 0.8 (0.69)  (n=163) |
| Scale score of EHP-30 at baseline, mean (SD) | | |  |  |  |  |
| Pain | 27.3 (20.99) | 27.3 (19.43) | 29.0 (20.12) | 27.0 (20.15) | 23.7 (18.77) | 26.9 (19.90) |
| Control and powerlessness | 25.0 (21.78) | 30.4 (23.57) | 26.6 (22.00) | 28.9 (24.06) | 25.1 (21.73) | 27.1 (22.57) |
| Emotional well-being | 20.7 (20.28) | 25.3 (19.72) | 21.0 (18.25) | 21.4 (20.08) | 21.9 (20.43) | 22.0 (19.70) |
| Social support | 16.1 (17.42) | 21.8 (21.83) | 15.8 (18.71) | 16.9 (21.42) | 16.9 (20.54) | 17.4 (19.97) |
| Self-image | 15.5 (16.63) | 18.0 (19.24) | 14.8 (17.98) | 16.3 (21.93) | 19.2 (22.22) | 16.7 (19.54) |
| Proportion of days with use of analgesics at baseline, %, mean (SD) | | | |  |  |  |
|  | 12.5 (12.40) | 13.0 (16.10) | 12.6 (15.03) | 12.4 (14.64) | 10.7 (12.43) | 12.3 (14.14) |

B&B score: Biberoglu and Behrman score; BMI: body mass index; EHP-30: Endometriosis Health Profile-30; M-B&B score: modified B&B score; SD: standard deviation; VAS: visual analog scale
