# Supplementary material for: Relugolix, an oral gonadotropin-releasing hormone (GnRH) receptor antagonist, in women with endometriosis-associated pain: phase 2 safety and efficacy 24-week results
Source: BMC Womens Health. 2021 Jun 21;21:250. doi: 10.1186/s12905-021-01393-3 (PMC8218467; doi:10.1186/s12905-021-01393-3)
Supplement: Supplementary file 2 — Additional file 2. Change from baseline in mean of M-B&B score for pelvic pain, dysmenorrhea, and dyspareunia by visit. [file 12905_2021_1393_MOESM2_ESM.docx]

**Additional file 2** Change from baseline in mean of M-B&B score for pelvic pain, dysmenorrhea, and dyspareunia by visit

| Variable | Relugolix 10 mg | Relugolix 20 mg | Relugolix 40 mg | Leuprorelin | Placebo |
| --- | --- | --- | --- | --- | --- |
| Change in mean score from baseline for pelvic pain, mean (SD) | | | |  |  |
| Days 1–28, n | 103 | 100 | 102 | 79 | 97 |
|  | −0.1 (0.25) | −0.1 (0.33) | −0.1 (0.30) | −0.2 (0.34) | −0.1 (0.33) |
| Days 29–56, n | 103 | 99 | 101 | 78 | 96 |
|  | −0.2 (0.31) | −0.2 (0.35) | −0.3 (0.35) | −0.3 (0.45) | −0.2 (0.38) |
| Days 57–84, n | 101 | 94 | 101 | 77 | 94 |
|  | −0.2 (0.30) | −0.2 (0.45) | −0.3 (0.40) | −0.4 (0.46) | −0.2 (0.35) |
| Days 85–112, n | 84 | 78 | 89 | 69 | 77 |
|  | −0.2 (0.34) | −0.2 (0.43) | −0.4 (0.39) | −0.5 (0.43) | −0.2 (0.40) |
| Days 113–140, n | 84 | 77 | 89 | 68 | 75 |
|  | −0.2 (0.36) | −0.3 (0.38) | −0.4 (0.49) | −0.5 (0.45) | −0.2 (0.38) |
| Days 141–168, n | 80 | 77 | 88 | 63 | 71 |
|  | −0.3 (0.37) | −0.3 (0.39) | −0.4 (0.45) | −0.5 (0.46) | −0.2 (0.36) |
| End of treatment, n | 103 | 100 | 103 | 79 | 97 |
|  | −0.3 (0.36) | −0.3 (0.39) | −0.4 (0.45) | −0.5 (0.49) | −0.2 (0.39) |
| Change in mean score from baseline for dysmenorrhea, mean (SD) | | | |  |  |
| Days 1–28, n | 103 | 100 | 103 | 80 | 97 |
|  | −0.3 (0.50) | −0.4 (0.53) | −0.4 (0.52) | −0.3 (0.54) | −0.2 (0.45) |
| Days 29–56, n | 103 | 99 | 101 | 78 | 96 |
|  | −0.4 (0.61) | −0.8 (0.68) | −1.2 (0.50) | −1.2 (0.51) | −0.2 (0.48) |
| Days 57–84, n | 101 | 94 | 101 | 77 | 95 |
|  | −0.5 (0.66) | −0.8 (0.66) | −1.2 (0.48) | −1.2 (0.47) | −0.2 (0.50) |
| Days 85–112, n | 84 | 78 | 89 | 69 | 77 |
|  | −0.5 (0.65) | −0.7 (0.66) | −1.2 (0.47) | −1.2 (0.48) | −0.2 (0.50) |
| Days 113–140, n | 84 | 77 | 89 | 68 | 75 |
|  | −0.6 (0.68) | −0.7 (0.64) | −1.2 (0.45) | −1.2 (0.48) | −0.2 (0.53) |
| Days 141–168, n | 80 | 77 | 88 | 63 | 71 |
|  | −0.5 (0.69) | −0.8 (0.61) | −1.2 (0.48) | −1.2 (0.49) | −0.2 (0.52) |
| End of treatment, n | 103 | 100 | 103 | 80 | 97 |
|  | −0.5 (0.67) | −0.8 (0.65) | −1.1 (0.50) | −1.2 (0.47) | −0.2 (0.55) |
| Change in mean score from baseline for deep dyspareunia, mean (SD) | | | |  |  |
| Days 1–28, n | 39 | 39 | 33 | 21 | 37 |
|  | 0.1 (0.41) | 0.1 (0.56) | 0.1 (0.46) | −0.2 (0.63) | −0.1 (0.34) |
| Days 29–56, n | 40 | 40 | 32 | 17 | 31 |
|  | 0.0 (0.51) | 0.0 (0.63) | −0.1 (0.43) | −0.2 (0.46) | −0.1 (0.41) |
| Days 57–84, n | 41 | 33 | 33 | 18 | 30 |
|  | −0.1 (0.49) | −0.1 (0.57) | −0.1 (0.51) | −0.3 (0.52) | −0.1 (0.34) |
| Days 85–112, n | 28 | 28 | 22 | 19 | 22 |
|  | −0.2 (0.35) | −0.2 (0.59) | −0.1 (0.55) | −0.2 (0.45) | 0.0 (0.43) |
| Days 113–140, n | 28 | 26 | 25 | 13 | 26 |
|  | −0.2 (0.41) | −0.2 (0.51) | −0.2 (0.32) | −0.3 (0.51) | −0.1 (0.30) |
| Days 141–168, n | 28 | 26 | 22 | 14 | 16 |
|  | −0.2 (0.42) | −0.2 (0.54) | −0.2 (0.32) | −0.2 (0.55) | 0.0 (0.42) |
| End of treatment, n | 40 | 34 | 31 | 18 | 29 |
|  | −0.2 (0.47) | −0.2 (0.49) | −0.1 (0.43) | −0.2 (0.56) | 0.0 (0.38) |

M-B&B score: modified Biberoglu and Behrman score; SD: standard deviation
