# Supplementary material for: Relugolix, an oral gonadotropin-releasing hormone (GnRH) receptor antagonist, in women with endometriosis-associated pain: phase 2 safety and efficacy 24-week results
Source: BMC Womens Health. 2021 Jun 21;21:250. doi: 10.1186/s12905-021-01393-3 (PMC8218467; doi:10.1186/s12905-021-01393-3)
Supplement: Supplementary file 3 — Additional file 3. Change from baseline in mean of B&B score for pelvic pain, dysmenorrhea, and dyspareunia by visit. [file 12905_2021_1393_MOESM3_ESM.docx]

**Additional file 3** Change from baseline in mean of B&B score for pelvic pain, dysmenorrhea, and dyspareunia by visit

| Variable | Relugolix 10 mg | Relugolix 20 mg | Relugolix 40 mg | Leuprorelin | Placebo |
| --- | --- | --- | --- | --- | --- |
| Change in mean score from baseline for pelvic pain, mean (SD) | | | |  |  |
| Week 4, n | 103 | 99 | 102 | 80 | 96 |
|  | −0.5 (0.79) | −0.5 (0.84) | −0.5 (0.78) | −0.7 (0.76) | −0.3 (0.79) |
| Week 8, n | 103 | 96 | 101 | 78 | 95 |
|  | −0.6 (0.88) | −0.6 (0.84) | −0.7 (0.88) | −0.8 (0.81) | −0.5 (0.78) |
| Week 12, n | 101 | 92 | 101 | 75 | 93 |
|  | −0.6 (0.76) | −0.8 (0.94) | −0.9 (0.84) | −1.1 (0.73) | −0.5 (0.75) |
| Week 16, n | 84 | 78 | 89 | 69 | 75 |
|  | −0.7 (0.83) | −0.9 (0.80) | −1.0 (0.78) | −1.2 (0.71) | −0.6 (0.78) |
| Week 20, n | 81 | 77 | 87 | 64 | 74 |
|  | −0.7 (0.78) | −0.8 (0.83) | −1.0 (0.84) | −1.2 (0.69) | −0.6 (0.81) |
| Week 24, n | 79 | 74 | 87 | 61 | 68 |
|  | −0.8 (0.79) | −0.9 (0.85) | −1.0 (0.86) | −1.2 (0.72) | −0.6 (0.85) |
| Change in mean score from baseline for dysmenorrhea, mean (SD) | | | |  |  |
| Week 4, n | 103 | 99 | 102 | 80 | 96 |
|  | −0.7 (0.85) | −0.8 (0.86) | −0.7 (0.90) | −1.0 (0.83) | −0.3 (0.64) |
| Week 8, n | 103 | 96 | 101 | 78 | 95 |
|  | −1.0 (0.92) | −1.5 (0.87) | −2.0 (0.64) | −2.0 (0.57) | −0.4 (0.71) |
| Week 12, n | 101 | 92 | 101 | 75 | 93 |
|  | −1.0 (0.93) | −1.5 (0.91) | −2.0 (0.51) | −2.1 (0.50) | −0.4 (0.76) |
| Week 16, n | 84 | 78 | 89 | 69 | 75 |
|  | −1.1 (0.90) | −1.5 (0.91) | −2.0 (0.54) | −2.1 (0.48) | −0.4 (0.70) |
| Week 20, n | 81 | 77 | 87 | 64 | 74 |
|  | −1.1 (0.91) | −1.4 (0.94) | −2.0 (0.56) | −2.1 (0.48) | −0.4 (0.82) |
| Week 24, n | 79 | 74 | 87 | 61 | 68 |
|  | −1.0 (0.87) | −1.5 (0.94) | −2.0 (0.61) | −2.1 (0.49) | −0.3 (0.64) |
| Change in mean score from baseline for dyspareunia, mean (SD) | | | |  |  |
| Week 4, n | 39 | 38 | 33 | 22 | 36 |
|  | 0.1 (0.65) | 0.0 (0.68) | 0.0 (0.53) | −0.5 (0.86) | −0.2 (0.71) |
| Week 8, n | 39 | 41 | 31 | 17 | 30 |
|  | −0.1 (0.70) | −0.2 (0.77) | 0.0 (0.60) | −0.5 (0.87) | −0.3 (0.69) |
| Week 12, n | 41 | 35 | 33 | 19 | 31 |
|  | −0.2 (0.68) | −0.2 (0.81) | −0.1 (0.60) | −0.6 (0.68) | −0.2 (0.72) |
| Week 16, n | 30 | 29 | 23 | 19 | 23 |
|  | −0.2 (0.57) | −0.4 (0.68) | −0.1 (0.67) | −0.5 (0.77) | 0.0 (0.60) |
| Week 20, n | 27 | 26 | 25 | 14 | 25 |
|  | −0.3 (0.66) | −0.5 (0.58) | −0.2 (0.55) | −0.6 (0.65) | −0.2 (0.72) |
| Week 24, n | 28 | 25 | 22 | 13 | 17 |
|  | −0.3 (0.67) | −0.4 (0.70) | −0.1 (0.43) | −0.5 (0.88) | −0.2 (0.83) |
| Change in mean score from baseline for pelvic tenderness, mean (SD) | | | |  |  |
| Week 4, n | 103 | 99 | 102 | 80 | 96 |
|  | −0.3 (0.74) | −0.4 (0.77) | −0.4 (0.69) | −0.5 (0.73) | −0.3 (0.63) |
| Week 8, n | 103 | 96 | 101 | 78 | 95 |
|  | −0.4 (0.75) | −0.6 (0.78) | −0.7 (0.81) | −0.7 (0.66) | −0.4 (0.69) |
| Week 12, n | 101 | 92 | 101 | 75 | 93 |
|  | −0.5 (0.73) | −0.7 (0.79) | −0.9 (0.89) | −0.8 (0.78) | −0.5 (0.80) |
| Week 16, n | 84 | 78 | 89 | 69 | 75 |
|  | −0.6 (0.83) | −0.7 (0.84) | −0.8 (0.78) | −1.0 (0.80) | −0.5 (0.68) |
| Week 20, n | 81 | 77 | 87 | 64 | 74 |
|  | −0.6 (0.83) | −0.8 (0.83) | −1.0 (0.87) | −1.0 (0.82) | −0.6 (0.78) |
| Week 24, n | 79 | 74 | 87 | 61 | 68 |
|  | −0.7 (0.83) | −0.8 (0.79) | −1.0 (0.92) | −1.1 (0.78) | −0.6 (0.74) |
| Change in mean score from baseline for induration, mean (SD) | | | |  |  |
| Week 4, n | 103 | 99 | 102 | 80 | 96 |
|  | −0.2 (0.66) | −0.4 (0.72) | −0.2 (0.63) | −0.3 (0.56) | −0.2 (0.50) |
| Week 8, n | 103 | 96 | 101 | 78 | 95 |
|  | −0.4 (0.64) | −0.6 (0.81) | −0.6 (0.80) | −0.5 (0.77) | −0.3 (0.56) |
| Week 12, n | 101 | 92 | 101 | 75 | 93 |
|  | −0.5 (0.69) | −0.6 (0.79) | −0.7 (0.82) | −0.7 (0.81) | −0.4 (0.65) |
| Week 16, n | 84 | 78 | 89 | 69 | 75 |
|  | −0.6 (0.78) | −0.7 (0.81) | −0.7 (0.82) | −0.8 (0.79) | −0.5 (0.60) |
| Week 20, n | 81 | 77 | 87 | 64 | 74 |
|  | −0.6 (0.77) | −0.7 (0.86) | −0.8 (0.82) | −0.8 (0.80) | −0.4 (0.72) |
| Week 24, n | 79 | 74 | 87 | 61 | 68 |
|  | −0.6 (0.81) | −0.7 (0.85) | −0.8 (0.81) | −0.8 (0.82) | −0.5 (0.72) |

B&B score: Biberoglu and Behrman score; SD: standard deviation
