# Supplementary material for: Relugolix, an oral gonadotropin-releasing hormone (GnRH) receptor antagonist, in women with endometriosis-associated pain: phase 2 safety and efficacy 24-week results
Source: BMC Womens Health. 2021 Jun 21;21:250. doi: 10.1186/s12905-021-01393-3 (PMC8218467; doi:10.1186/s12905-021-01393-3)
Supplement: Supplementary file 4 — Additional file 4. Results of other endpoints. [file 12905_2021_1393_MOESM4_ESM.docx]

**Additional file 4** Results of other endpoints

| Variable | Relugolix  10 mg | Relugolix  20 mg | Relugolix  40 mg | Leuprorelin | Placebo |
| --- | --- | --- | --- | --- | --- |
| Change in proportion of days with use of analgesics from baseline, %, mean (SD) | | | | | |
| Days 1–28, n | 103 | 100 | 103 | 80 | 97 |
|  | −6.5 (9.04) | −4.4 (10.72) | −4.8 (11.80) | −4.0 (9.66) | −1.1 (10.31) |
| Days 29–56, n | 103 | 99 | 101 | 78 | 96 |
|  | −6.0 (10.52) | −7.5 (12.30) | −10.3 (12.57) | −7.7 (12.45) | −1.5 (12.87) |
| Days 57–84, n | 101 | 94 | 101 | 77 | 95 |
|  | −6.8 (11.10) | −7.2 (14.94) | −10.3 (13.48) | −8.4 (13.69) | −2.2 (10.67) |
| Days 85–112, n | 84 | 78 | 89 | 69 | 77 |
|  | −6.7 (11.92) | −7.9 (13.84) | −11.4 (14.43) | −10.2 (11.38) | −1.9 (12.68) |
| Days 113–140, n | 84 | 77 | 89 | 68 | 75 |
|  | −6.5 (11.21) | −7.4 (13.63) | −10.5 (15.98) | −10.1 (13.00) | −1.8 (12.36) |
| Days 141–168, n | 80 | 77 | 88 | 63 | 71 |
|  | −6.9 (10.39) | −7.8 (15.19) | −10.8 (14.94) | −11.9 (13.33) | −1.5 (10.92) |
| End of treatment, n | 103 | 100 | 103 | 80 | 97 |
|  | −6.3 (9.82) | −7.4 (14.59) | −10.0 (14.21) | −10.2 (13.10) | −0.6 (10.25) |
| Change in EHP-30 scores from baseline, mean (SD) | | | |  |  |
| Week 12, n | 101 | 92 | 101 | 75 | 93 |
| Pain | −18.3 (19.76) | −17.8 (20.36) | −25.3 (20.87) | −23.3 (20.50) | −5.6 (18.99) |
| Control and powerlessness | −13.7 (18.71) | −14.6 (23.59) | −17.2 (22.48) | −19.8 (23.35) | −8.2 (18.74) |
| Emotional well-being | −8.3 (16.44) | −8.9 (18.62) | −10.4 (17.77) | −8.9 (17.34) | −6.3 (14.48) |
| Social support | −6.6 (10.29) | −8.4 (16.95) | −6.8 (15.19) | −6.8 (16.47) | −3.2 (14.59) |
| Self-image | −5.5 (11.56) | −6.3 (14.90) | −8.4 (16.18) | −6.2 (16.44) | −3.9 (16.42) |
| Week 24, n | 79 | 74 | 87 | 61 | 68 |
| Pain | −17.0 (20.29) | −20.6 (19.65) | −25.9 (19.90) | −26.4 (20.34) | −5.4 (18.42) |
| Control and powerlessness | −14.0 (17.50) | −20.0 (21.88) | −20.9 (21.68) | −24.8 (23.84) | −6.9 (15.85) |
| Emotional well-being | −8.4 (15.92) | −15.4 (17.86) | −13.3 (16.32) | −12.4 (18.33) | −6.7 (17.70) |
| Social support | −7.5 (10.84) | −13.4 (17.06) | −10.3 (17.11) | −10.5 (17.92) | −3.2 (16.61) |
| Self-image | −5.9 (12.81) | −10.6 (15.26) | −9.7 (17.74) | −9.4 (15.55) | −5.4 (15.42) |

EHP-30: Endometriosis Health Profile-30; SD: standard deviation
