# Supplementary material for: Relugolix, an oral gonadotropin-releasing hormone (GnRH) receptor antagonist, in women with endometriosis-associated pain: phase 2 safety and efficacy 24-week results
Source: BMC Womens Health. 2021 Jun 21;21:250. doi: 10.1186/s12905-021-01393-3 (PMC8218467; doi:10.1186/s12905-021-01393-3)
Supplement: Supplementary file 5 — Additional file 5. Summary of pharmacodynamic parameters at each assessment point. [file 12905_2021_1393_MOESM5_ESM.docx]

**Additional file 5** Summary of pharmacodynamic parameters at each assessment point

|  | Relugolix | | | Leuprorelin | Placebo |
| --- | --- | --- | --- | --- | --- |
|  | 10 mg | 20 mg | 40 mg |  |  |
| E_2_ (pg/mL)^a^, median | | | | | |
| Baseline, n | 103 | 100 | 103 | 80 | 97 |
|  | 45.0 | 41.0 | 44.0 | 42.0 | 41.0 |
| Week 2, n | 103 | 100 | 103 | 80 | 97 |
|  | 56.0 | 26.5 | 0.0 | 12.0 | 170.0 |
| Week 4, n | 103 | 99 | 101 | 79 | 96 |
|  | 48.0 | 24.0 | 0.0 | 0.0 | 56.0 |
| Week 8, n | 103 | 96 | 101 | 78 | 95 |
|  | 50.0 | 25.0 | 0.0 | 0.0 | 94.0 |
| Week 12, n | 101 | 92 | 101 | 75 | 93 |
|  | 62.0 | 24.5 | 0.0 | 0.0 | 112.0 |
| Week 24, n | 79 | 74 | 87 | 61 | 68 |
|  | 39.0 | 26.5 | 0.0 | 0.0 | 94.0 |
| Follow-up, n | 83 | 77 | 89 | 69 | 77 |
|  | 88.0 | 107.0 | 137.0 | 11.0 | 122.0 |
| LH (mIU/mL), median | | | | | |
| Baseline, n | 103 | 100 | 103 | 80 | 97 |
|  | 3.67 | 3.59 | 3.37 | 3.47 | 3.65 |
| Week 2, n | 103 | 100 | 103 | 80 | 97 |
|  | 5.25 | 3.13 | 0.36 | 2.20 | 4.60 |
| Week 4, n | 103 | 99 | 101 | 79 | 96 |
|  | 2.98 | 2.23 | 0.29 | 0.56 | 3.90 |
| Week 8, n | 103 | 96 | 101 | 78 | 95 |
|  | 3.55 | 2.58 | 0.39 | 0.24 | 3.92 |
| Week 12, n | 101 | 92 | 101 | 75 | 93 |
|  | 4.05 | 2.78 | 0.53 | 0.21 | 4.24 |
| Week 24, n | 79 | 74 | 87 | 61 | 68 |
|  | 3.60 | 2.79 | 0.88 | 0.18 | 4.24 |
| Follow-up, n | 83 | 77 | 89 | 69 | 77 |
|  | 4.96 | 4.52 | 4.47 | 0.30 | 4.38 |
| FSH (mIU/mL), median | | | | | |
| Baseline, n | 103 | 100 | 103 | 80 | 97 |
|  | 6.64 | 6.03 | 6.24 | 6.38 | 6.24 |
| Week 2, n | 103 | 100 | 103 | 80 | 97 |
|  | 5.93 | 5.88 | 3.39 | 1.61 | 3.80 |
| Week 4, n | 103 | 99 | 101 | 79 | 96 |
|  | 5.09 | 4.72 | 2.32 | 1.94 | 5.65 |
| Week 8, n | 103 | 96 | 101 | 78 | 95 |
|  | 5.34 | 4.64 | 2.25 | 3.03 | 5.19 |
| Week 12, n | 101 | 92 | 101 | 75 | 93 |
|  | 5.51 | 4.81 | 2.39 | 3.42 | 5.19 |
| Week 24, n | 79 | 74 | 87 | 61 | 68 |
|  | 5.74 | 5.53 | 2.50 | 3.79 | 5.03 |
| Follow-up, n | 83 | 77 | 89 | 69 | 77 |
|  | 4.63 | 4.41 | 3.67 | 4.99 | 4.85 |
| P (ng/mL), median | | | | | |
| Baseline, n | 103 | 100 | 103 | 80 | 97 |
|  | 0.35 | 0.29 | 0.33 | 0.34 | 0.34 |
| Week 2, n | 103 | 100 | 103 | 80 | 97 |
|  | 0.34 | 0.27 | 0.25 | 0.29 | 5.45 |
| Week 4, n | 103 | 99 | 101 | 79 | 96 |
|  | 0.44 | 0.35 | 0.26 | 0.24 | 0.38 |
| Week 8, n | 103 | 96 | 101 | 78 | 95 |
|  | 0.49 | 0.30 | 0.24 | 0.27 | 0.68 |
| Week 12, n | 101 | 92 | 101 | 75 | 93 |
|  | 0.39 | 0.36 | 0.24 | 0.24 | 0.58 |
| Week 24, n | 79 | 74 | 87 | 61 | 68 |
|  | 0.40 | 0.34 | 0.22 | 0.27 | 0.53 |
| Follow-up, n | 83 | 77 | 89 | 69 | 77 |
|  | 0.80 | 1.01 | 6.24 | 0.28 | 0.54 |

^a^The lower limit of quantification of E_2_ was 10 pg/mL
